# Hydrazine-Selective Fluorescent Turn-On Probe Based on Ortho-Methoxy-Methyl-Ether (*o*-MOM) Assisted Retro-aza-Henry Type Reaction

**DOI:** 10.3390/s19204525

**Published:** 2019-10-17

**Authors:** Yuna Jung, Nam Kyoo Park, Jae Seung Kang, Dokyoung Kim

**Affiliations:** 1 Department of Biomedical Science, Graduate School, Kyung Hee University, Seoul 02447, Korea; jungpeng159@gmail.com (Y.J.); namkyupk@gmail.com (N.K.P.); 2 Laboratory of Vitamin C and Antioxidant Immunology, Department of Anatomy and Cell Biology, Seoul National University, College of Medicine, Seoul 03080, Korea; 3 Institute of Allergy and Clinical Immunology, Seoul National University Medical Research Center, Seoul 03080, Korea; 4 Department of Anatomy and Neurobiology, College of Medicine, Kyung Hee University, Seoul 02447, Korea; 5 Center for Converging Humanities, Kyung Hee University, Seoul 02447, Korea; 6 Medical Research Center for Bioreaction to Reactive Oxygen Species and Biomedical Science Institute, School of Medicine, Graduate School, Kyung Hee University, Seoul 02447, Korea

**Keywords:** fluorescent probe, off-on response, hydrazine detection, dipolar fluorophore, molecular rotor

## Abstract

Hydrazine (N_2_H_4_) is one of the most widely used industrial chemicals that can be utilized as a precursor of pesticides, pharmaceutics, and rocket propellant. Due to its biological and environmental toxicity with potential health risks, various sensing tools have been developed. Among them, fluorescence-based molecular sensing systems have been highlighted due to its simple-operation, high selectivity and sensitivity, and biocompatibility. In our recent report, we disclosed a ratiometric type fluorescent probe, called **HyP-1**, for the detection of hydrazine, which is based on *ortho*-methoxy-methyl-ether (*o*-MOM) moiety assisted hydrazone-formation of the donor (D)-acceptor (A) type naphthaldehyde backbone. As our follow-up research, we disclose a turn-on type fluorescent probe, named **HyP-2**, as the next-generation hydrazine probe. The sensing rational of **HyP-2** is based on the *o*-MOM assisted retro-aza-Henry type reaction. The dicyanovinyl moiety, commonly known as a molecular rotor, causes significant emission quenching of a fluorescent platform in aqueous media, and its cleavage with hydrazone-formation, which induces a significant fluorescence enhancement. The high selectivity and sensitivity of **HyP-2** shows practical explicabilities, including real-time paper strip assay, vapor test, soil analysis, and real water assay. We believe its successful demonstrations suggest further applications into a wide variety of fields.

## 1. Introduction

We have highlighted the development of new fluorometric platforms, for the detection of toxic chemical species, due to its close relationship with many biological and environmental processes [1,2,3]. Hydrazine (N_2_H_4_) is a well-known industrial pnictogen hydride chemical, which is widely used in pharmaceutical products such as catalysts, as a propellant and agriculture pesticide [4,5,6,7]. However, hydrazine has shown toxicity to organisms and within various environments [8,9,10]. It can cause crucial damage to the human central nervous system (CNS), liver, lungs, and kidneys. Recognizing the importance of sensing hydrazine, many analytical methods have been introduced, mainly instrument-dependent tools, such as mass spectrometric analysis, electrochemical approach, and chromatography [11,12,13]. Fluorescent techniques have also been developed using chemistry-based tools, owing to their simple operation, high selectivity and responsiveness, and high compatibility toward biological and environmental analytes [14,15].

Thus far, many fluorescent probes that can sense hydrazine have been reported [16]. The working mechanisms of known probes are mostly based on (i) hydrazine-triggered sensing moiety cleavage, (ii) hydrazine-addition induced sensing moiety transformation. However, previously reported fluorescent probes with the following mechanism still have drawbacks, such as a long reaction time, low selectivity and sensitivity, and organic solvent containing sensing media [17,18]. As fluorescence-based sensing technology advances, the following options need to be considered for the hydrazine sensing; (i) signal response in various sensing media, including pure water, with high selectivity and sensitivity, (ii) signal monitoring without using special instruments. However, the designing of new sensing moiety and fluorescent probes that address all the issues above is still very challenging.

**Figure 1:**
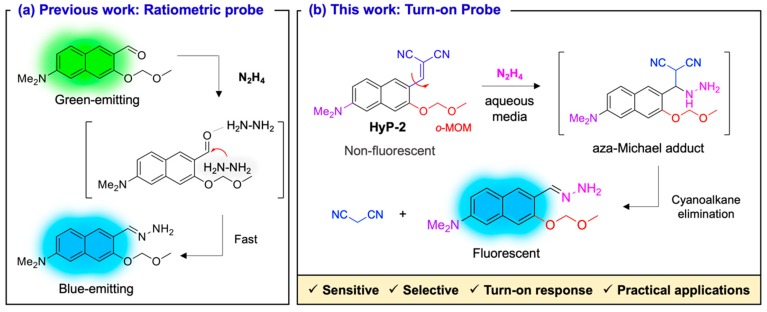
(**a**) Previous work: ratiometric type fluorescent probe (**HyP-1**) for the detection of hydrazine. (**b**) Current work: turn-on type fluorescent probe (**HyP-2**) for the detection of hydrazine based on *ortho*-methoxy-methyl-ether (*o*-MOM) assisted Retro-aza-Henry type reaction. Schematic illustration of the sensing strategy and merits of **HyP-2** are briefly described.

In our previous work, a ratiometric type of fluorescent probe, **HyP-1**, was reported for the detection of hydrazine, which was based on *ortho*-methoxy-methyl-ether (*o*-MOM) moiety assisted fast hydrazone-formation within the donor (D)-acceptor (A) type dipolar naphthaldehyde backbone (Figure 1a) [18]. In this report, we reveal a newly developed turn-on type fluorescent probe, **HyP-2**, which is based on the *o*-MOM assisted retro-aza-Henry type reaction [19]; hydrazine addition, aza-Michal adduct formation, and cyanoalkane elimination (Figure 1b). We verified the high sensitivity (limit of detection (LOD) value around 0.05 ppb (1.56 nM)), selectivity, and turn-on response of **HyP-2** for the detection of hydrazine as a follow-up research to our previous work. For the practical applications of **HyP-2**, we successfully demonstrated the sensing of hydrazine using a paper strip, and monitoring of hydrazine in the environmental samples (soil, lake water, river water, sea water, tap water, and commercial bottled drinking water). Through this research, both turn-on and ratiometric type hydrazine-probe rounded out from the same fluorescent platform.

## 2. Materials and Methods

### 2.1. Materials

The chemical reagents were purchased from Aldrich (St. Louis, MO, USA), TCI (Tokyo, Japan), Alfa Aesar (Ward Hill, MA, USA), Samchun (Seoul, Korea), and Daejung Chemicals (Siheung, Korea). Metal ions and amino acid (Alfa Aesar, Aldrich, Daejung, ≥ 97% purity): CaCl_2_, CdCl_2_, CuCl_2_, FeCl_3_, KCl, MgCl_2_, NaCl, NaCN, NaHSO_3_, NaN_3_, NaOAc, NaOH, NaSH, NiCl_2_, ZnCl_2_, L-glutathione (GSH), L-glutamine (Glu), L-cysteine (Cys), DL-homocysteine (Hcy), L-glutamine (Glu), L-lysine (Lys), and L-aspartic acid (Asp). Hydrazine solution (35% in deionized water) was purchased from Aldrich (St. Louis, MO, USA). The pH range was 4–9, including biological pH (7.4) for the pH screening. The pH buffers were purchased from Daejung Chemicals (Siheung, Gyeonggi-do, Korea). The cellulose-based filter paper (Whatman^TM^, Cat 1002-105, Maidstone, UK) was purchased for paper strip applications. The cell culture dish (SPL Life Science, #20060, 60 mm × 15 mm, Pocheon, Gyeonggi-do, Rep. of Korea) and soils (clay, sand, and field soil; Goyang, Gyeonggi-do, Science Love, Korea) were purchased for soil analysis applications. Commercially available reagents and solvents (anhydrous) were used without further purification. Chemical reactions were performed under argon atmosphere. Thin-layer chromatography (TLC) was conducted using pre-coated silica gel (60F-254 glass plates, Merck, Darmstadt, Germany). Real water samples were collected or purchased: (i) lake water (Jemyoung lake, Seoul, Rep. of Korea), (ii) river water (Han-river, Seoul, Rep. of Korea), (iii) sea water (Oido, Yellow Sea, Siheung, Gyeonggi-do, Rep. of Korea), (iv) tap water (Kyung Hee University, College of Medicine Building, Seoul, Rep. of Korea), and (v) commercial bottled drinking water (Lotte ICIS (Gyeongsangbuk-do), (vi) Jeju Samdasoo (Jeju-do), 500 mL bottle, Rep. of Korea).

### 2.2. Synthesis

**Figure 2:**
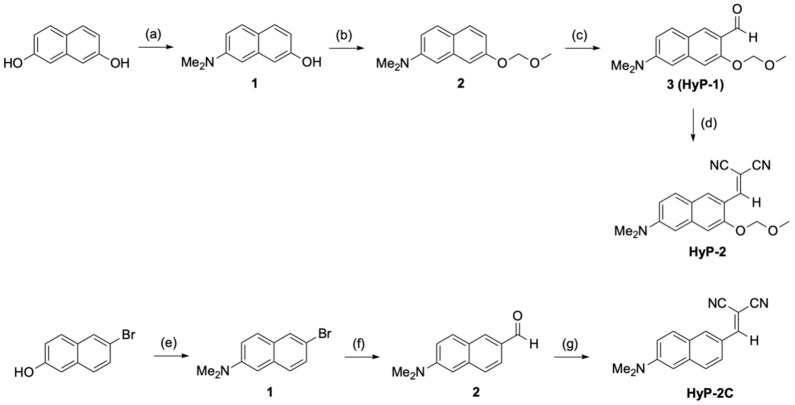
Synthetic schemes for **HyP-2** and **HyP-2C** (control compound; no *o*-MOM). (**a**) Na_2_S_2_O_5_, Me_2_NH, DI H_2_O, 150 °C, 3 h, 60%; (**b**) NaH, THF, CH_3_OCH_2_Cl, −15 °C, 7 h, 95%; (**c**) *t*-BuLi, diethyl ether, DMF, −15 °C, 2 h, 52%. (**d**) CH_2_(CN)_2_, piperidine, EtOH, 25 °C, 30 min, 71%; (**e**) Na_2_S_2_O_5_, Me_2_NH, DI H_2_O, 150 °C, 72 h, 70%;(**f**) *n*-BuLi, THF, DMF, -78 to -30 °C, 0.5 h, 85%; (**g**) CH_2_(CN)_2_, piperidine, EtOH, 25 °C, 20 min, 90%.

**HyP-2** was prepared via Knoevenagel condensation between the intermediate (compound **3**; **HyP-1**) and malononitrile in the presence of piperidine catalyst (Figure 2). The key intermediate **3** was synthesized using the following reported method by our group [20]. Directed lithiation and formylation are key steps of the synthesis. Overall yield of the 4 steps was 71% (**HyP-2**). **HyP-2C** was also prepared following the known protocols; 3 steps, 90% yield. [CAUTION: for directed lithiation step] *t*-BuLi is very reactive and fragile. The appropriate PPE (personal protective equipment) was used for careful preparation. The purity of synthesized **HyP-2** and **HyP-2c** was confirmed by ^1^H/^13^C NMR and high-resolution (HR) mass spectrometry analysis (See Supplementary Information).

### 2.3. UV/Vis Absorption and Emission Measurement

UV/Vis absorption spectra were acquired using a spectro-photometer (Agilent Technologies, Cary 8454, Santa Clara, CA, USA). Emission spectra was obtained by a spectro-fluorophotometer (SHIMADZU, RF-6000, Kyoto, Japan) with a 1 cm standard quartz cell (internal volume of 1 mL, 108-000-10-40 (10 mm), 108-F-10-40 (10 × 4 mm), Hellma Analytics, Müllheim, Germany). The absorption and fluorescence spectra were obtained at 10 µM concentration at 25 °C within given solvents. Photo-stability of **HyP-2** was recorded under continuous exposure of UV light (365 nm, 3 W, Rayman-RM104, Goyang, Gyeonggi-do, Korea) in deionized water (DI H_2_O) for 60 min at 25 °C. During the UV light exposure, UV/Vis absorption and fluorescence spectra changes were monitored at given time intervals (10 min). The maximum absorption wavelength was applied for the acquirement of fluorescence emission spectra. Quantum yield (Q.Y.) of hydrazone product were measured using 9,10-diphenylanthracene standard (Q.Y. = 0.88). The experiments with real water samples were carried out by following methods above.

### 2.4. NMR and Mass Analysis

^1^H NMR and ^13^C NMR spectra were obtained with Bruker AVANCE III 400 MHz (Bruker, Billerica, MA, USA). In the analyzed NMR spectra, the chemical shifts (δ) are described as ppm, multiplicity is indicated by s (singlet), *d* (doublet), *t* (triplet), *dd* (double of doublets), and *m* (multiplet). Spectra was referenced to residual DMSO (2.50 ppm) or chloroform (7.26 ppm) in ^1^H NMR. High-resolution mass spectrometer results were obtained on JEOL JMS-700 spectrometer (Tokyo, Japan) at the Korea Basic Science Center, Kyung-pook National University, and the values are reported in units of mass to charge (*m/z*).

### 2.5. Paper Strip Test

**HyP-2** solution in DMSO (30 μM) was sprayed three times to a cellulose-based filter paper and dried at 25 °C. **HyP-2** pre-treated paper was soaked in hydrazine solution (100 mM in deionized water) for 1 s and air-dried at 25 °C for 1 min. The fluorescence change of the strips was recorded using a digital camera (Sony, Alpha A5100, Tokyo, Japan) under UV light (365 nm).

### 2.6. Vapor Test

A cellulose-based filter paper (Whatman^TM^, Cat 1002-105, Maidstone, UK) was soaked into **HyP-2** solution (100 μM in DMSO) once and dried at 25 °C. The papers were attached to 20 mL vial caps, and then exposed to various vapors, including H_2_O, N_2_H_4_, HN(CH)_2_, H_2_CO, HCl, and H_2_CO/N_2_H_4_ with heating (~ 100 °C) for 30 s. The fluorescence changes of each paper strips were recorded using a digital camera under UV light (365 nm).

### 2.7. Soil Analysis

The hydrazine sensing application in the soil samples using **HyP-2** was conducted in two different types of experimental set. [Type 1] Three spoons of soils (sand, clay, and field; Science Love, Rep. of Korea) were transferred to the cell culture dishes. 3 mL of hydrazine solution in deionized water (100 mM) was treated to the culture dishes at 25 °C. Soils were then incubated for at 25 °C for 1 min. After incubation, 4 μL of **HyP-2** solution in DMSO (10 mM) was treated once in the middle of the culture dish, which included the soils incubated with the hydrazine solution. The fluorescence changes were monitored using a digital camera under UV light (365 nm). [Type 2] Three spoons of each soil were transferred to culture dishes. A plastic dish containing 100 μL of hydrazine solution (35% in DI H_2_O) was placed on the soils (the red-colored circle in Figure 8c). Then, **HyP-2** (30 μM) was sprayed 10 times towards each soil. The real-time fluorescence changes were recorded using a digital camera under UV light (365 nm).

## 3. Results and Discussion

### 3.1. Probe Design

Recently, our research team has focused on the development of a naphthalene-based donor (D)-acceptor (A) type of dipolar fluorescent dyes and its applications for the detection of metal ions, enzyme activity, amino acid, cell sub-organelles, disease biomarkers, and carcinogens [20,21,22,23,24,25]. During the research process, we found a fast hydrazone-formation between D-A type naphthaldehyde and hydrazine by *o*-MOM assistant, and we utilized this property in developing a fluorescent probe, **HyP-1 [18]**. As a follow-up research of **HyP-1**, we designed a new turn-on type of fluorescent probe, named **HyP-2**, which consisted of *o*-MOM and dicyanovinyl molecular rotor moiety that can be cleavaged by hydrazine (Figure 1). Although the ratiometric type probe has merits, in the substrate analysis, a turn-on probe is also preferred, mostly due to its directed signal response with low interference and large signal-to-noise ratio coming from the non-specific quenchers and desired substrate [26,27]. We expected the negligible fluorescence of **HyP-2** dipolar backbone due to the effect of polarity on charge redistribution in molecule within aqueous media [28,29,30]. The addition of hydrazine can induce the cleavage of the dicyanovinyl group [16] and cascade hydrazone-formation via retro-aza-Henry type reaction, and it could be accelerated by *o*-MOM moiety. Using this process, the fluorescence of **HyP-2** was recovered as a turn-on manner due to the elimination of non-radiative decay pathway. The approach, cleavage of the dicyanovinyl group, and its functionalization within the fluorescent probe for the detection of hydrazine have already been documented (Table S1), but this is the first time we have reported the *o*-MOM assisted fast hydrazone-formation.

With this rational, the **HyP-2** was prepared using 4-step reactions (Figure 2), and its hydrazine sensing ability was systematically analyzed with practical demonstrations as outlined below.

### 3.2. Photophysical Property Analysis and Hydrazine Sensing Study of HyP-2

**Figure 3:**
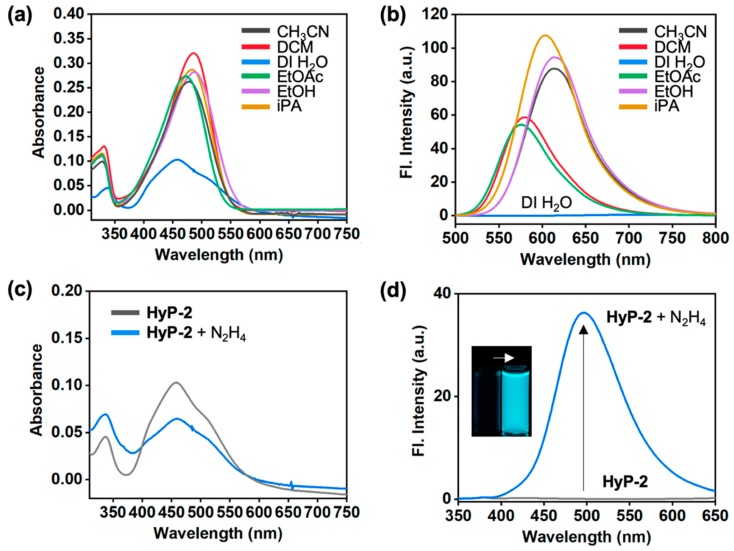
Photophysical properties and Hydrazine (N_2_H_4_) sensing properties of **HyP-2**. (**a**) Absorption and (**b**) emission spectra of **HyP-2** (10 μM) in various solvents. Solvents; CH_3_CN, acetonitrile; DCM, dichloromethane; DI H_2_O, deionized water; EtOAc, ethyl acetate; EtOH, ethanol; iPA, isopropanol. The emission spectra were obtained under excitation at the maximum wavelength of absorption within each solvent. (**c**) Absorption and (**d**) emission spectra of **HyP-2** (10 μM) after adding hydrazine (1 mM) in DI H_2_O, before (gray line) and after incubation at 25 °C for 60 min (blue line). Inset: A photo of **HyP-2** before and after treatment of hydrazine (1 mM) under UV light (365 nm). The emission spectra were obtained under excitation at the maximum wavelength of absorption (338 nm).

First, basic photophysical property of **HyP-2** was analyzed in various solvents (Figure 3, Figure S1, Table S2). In the UV/Vis spectra, **HyP-2** showed a main absorbance peak around 459–487 nm in organic solvents, as well as deionized water (DI H_2_O) (Figure 3a). In the emission spectra, **HyP-2** showed a maximum peak around 576–614 nm with solvatochromic shift, which is a typical phenomenon of dipolar dye [31], except DI H_2_O due to the non-radiative decay pathway generation from the molecular rotor moiety (Figure 3b).

**Figure 4:**
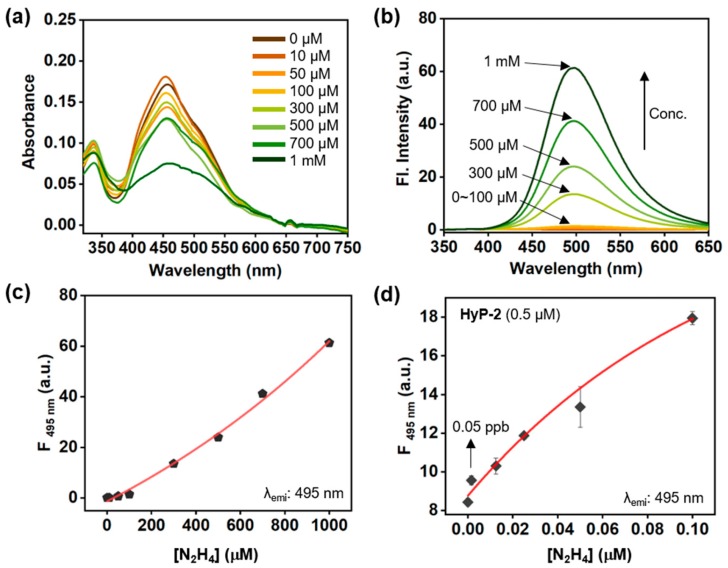
Hydrazine concentration-dependent absorption and emission spectra changes of **HyP-2**. (**a**) Absorption and (**b**) emission spectra change of **HyP-2** (10 μM) after adding hydrazine (0–100 equivalent; 0–1 mM) in DI H_2_O, analyzed after incubation for 60 min at 25 °C. (**c**) A plot of fluorescence intensity (λ_emi_: 495 nm) of **HyP-2** (10 μM) after adding hydrazine (1 mM) in DI H_2_O, analyzed after incubation for 60 min at 25 °C. (**d**) A plot of fluorescence intensity (λ_emi_: 495 nm) of **HyP-2** (10 μM) after adding hydrazine at low concentration (0–0.1 μM) in DI H_2_O, analyzed after incubation for 60 min at 25 °C. The emission spectra were obtained under excitation at the maximum wavelength of absorption within each concentration.

The absorption and emission spectra changes of **HyP-2** were monitored within DI H_2_O containing no organic co-solvent. No aggregation factor of **HyP-2** was monitored at given concentration (10 μM) (Figure S2). Upon treatment with hydrazine, **HyP-2** emitted a strong fluorescence at 495 nm, with changes of absorption intensity; an increment at 336 nm and a decrement at 459 nm, by producing hydrazone compound (Figure 3c,d) (quantum yield (Q.Y.) of product: 0.47, Figure S3). The generation of hydrazone compound was confirmed by spectrum analysis by comparing it with the known compound (**HyP-1** + N_2_H_4_) [23], ^1^H NMR analysis (Figure S4), and HR-mass spectrometry analysis (Supplementary Information, *m*/*z* = 273.1476, calc. = 273.1477). A good non-linear relationship between the fluorescence intensity of **HyP-2** and hydrazine concentration (0–1 mM in DI H_2_O) was observed (Figure 4). Fluorescence intensity analysis at low concentrations of hydrazine (below 0.1 μM), **HyP-2** gave a LOD value around 0.05 ppb (1.56 nM) according to a S/N (signal-to-noise) criteria ratio of more than three, which is 200 times lower than the concentration level set by U.S. EPA (10 ppb).

**Figure 5:**
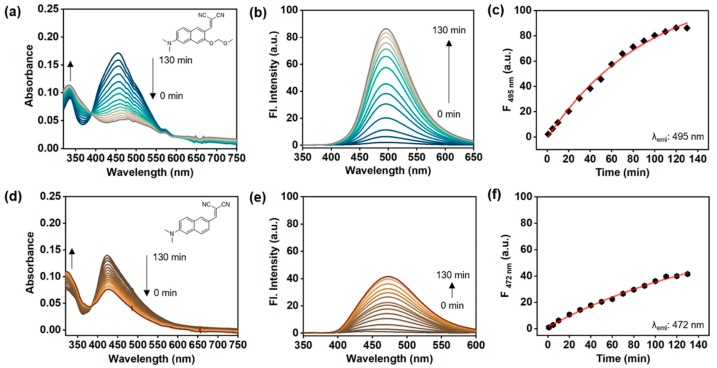
Time-dependent absorption and emission spectra change of **HyP-2** and **HyP-2C** (control compound). (**a**) Absorption and (**b**) emission change of **HyP-2** (10 μM) after adding hydrazine (1 mM) in DI H_2_O, analyzed after incubating for 0–130 min (10 min intervals) at 25 °C. (**c**) A plot of fluorescence intensity (λ_emi_: 495 nm) of **HyP-2** (10 μM) after adding hydrazine (1 mM) in DI H_2_O, analyzed after incubating for 0–130 min at 25 °C. (**d**) Absorption and (**e**) emission spectra change of **HyP-2C** (10 μM) after adding hydrazine (1 mM) in DI H_2_O, analyzed after incubation for 0–130 min (10 min intervals) at 25 °C. (**f**) A plot of fluorescence intensity (λ_emi_: 472 nm) of **HyP-2C** (10 μM) after adding hydrazine (1 mM) in DI H_2_O, and analyzed after incubating for 0–130 min at 25 °C. The emission spectra were obtained under excitation at the maximum wavelength of absorption.

To confirm the effect of the *o*-MOM moiety in the hydrazine sensing, we prepared a control compound, **HyP-2C**, which has no *o*-MOM moiety (see the structure and synthetic scheme in Figure 2), and checked the sensing ability towards hydrazine (1 mM) compared with **HyP-2**. The maximum absorption and emission wavelength of **HyP-2** and **HyP-2C** were measured around λ_abs_ 330–550 nm and λ_emi_ 400–650 nm ranges. **HyP-2** showed a slightly longer wavelength of absorption (λ_abs.max_ = 337 nm) and emission (λ_abs.max_ = 495 nm) than **HyP-2C** (λ_abs.max_ = 316 nm, λ_emi.max_ = 472 nm). In the time-course fluorescence analysis, **HyP-2** showed remarkable absorption changes and fluorescence enhancements after adding hydrazine (1 mM in DI H_2_O) within 5 min, and its fluorescence intensity gradually increased over 130 min (Figure 5a–c). In contrast, **HyP-2C** showed a slow response within the given conditions (Figure 5d–f). As a result of this direct comparison, we discovered that the *o*-MOM moiety is necessary in order to accelerate the cleavage of the dicyanovinyl group and hydrazone-formation.

### 3.3. Selectivity and pH Screening

**Figure 6:**
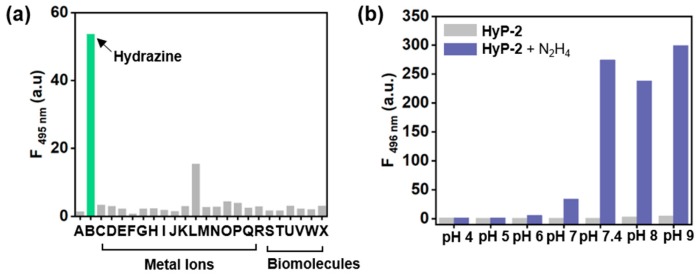
Sensing properties of **HyP-2**. (**a**) Fluorescence intensity (λ_emi_: 495 nm) of **HyP-2** (10 μM) after the addition of various metal ions/anions (30 eq) or biomolecules (30 eq) in DI H_2_O, analyzed after incubation for 60 min at 25 °C. Metal ions/anions; (A) **HyP-2**, (B) N_2_H_4_, (C) CaCl_2_, (D) CdCl_2_, (E) CuCl_2_, (F) FeCl_3_, (G) KCl, (H) MgCl_2_, (I) NaCl, (J) NaCl (anion), (K) NaCN, (L) NaHSO_3_, (M) NaN_3_, (N) NaOAc, (O) NaOH, (P) NaSH, (Q) NiCl_2_, (R) ZnCl_2_. Biomolecules; (S) Glu (glutamine), (T) GSH (glutathione), (U) Lys (lysine), (V) Cys (cysteine), (W) Hcy (homocysteine), and (X) Asp (aspartic acid). The emission spectra were obtained under excitation at 338 nm. (**b**) Fluorescence intensity (λ_emi_: 495 nm) of **HyP-2** (10 μM) after adding hydrazine (1 mM) in various pH buffers (pH 4, 5, 6, 7, 7.4, 8, 9), analyzed after incubating for 60 min at 25 °C. The emission spectra were obtained under excitation at the maximum absorption wavelength within each pH buffers.

The selectivity of **HyP-2** towards hydrazine was evaluated by monitoring the fluorescence intensity changes after adding hydrazine, metal ions, and biomolecules (Figure 6a, Figure S5). We observed a strong fluorescence enhancement of **HyP-2** towards hydrazine, and we recorded negligible responses toward the other metal ions and enzymes. An interference occurred only in the hydrogen sulfite (HSO^3-^, “L” in Figure 6a), which derived from the adduct formation between sulfite and dicyanovinyl moiety [32]. However, at an emission of 495 nm, **HyP-2** showed high selectivity towards hydrazine (36-times enhancement) over hydrogen sulfite (10 times), and the spectrum analysis also showed a clear distinction of hydrazine (Figure S5). In the pH-dependent (pH 4–9 including physiological pH 7.4) sensing assay, the optimal sensing behavior of **HyP-2** to hydrazine was at alkali pHs, pH 7.4–9, whose property is similar to **HyP-1** (Figure 6b, Figure S6). These results concluded that **HyP-2** can be used for selective and sensitive detection of hydrazine within environmental and biological samples. The photostability of **HyP-2** was monitored under the continuous irradiation of UV hand light (365 nm, 3 W) for 1 h. No significant fluorescence changes were observed under these given conditions, representing a high photostability of **HyP-2** (Figure S7).

### 3.4. Paper Strip Spray Assay

**Figure 7:**
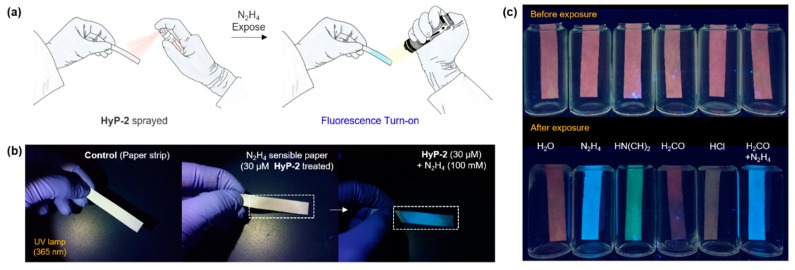
Paper strip spray application of **HyP-2**. (**a**) A schematic illustration of the paper strip sensing test. Protocol: (i) **HyP-2** (30 μM in DMSO) spraying three times; (ii) paper strip soaking in hydrazine solution. (iii) paper strip monitoring under UV light (365 nm). (**b**) Photos of paper strip (control), **HyP-2** treated paper strip (30 μM **HyP-2** sprayed), and N_2_H_4_-exposed paper strip (soaked into 100 mM N_2_H_4_ solution for 1 s). (**c**) Vapor test with the **HyP-2** pre-treated paper. Photos of **HyP-2** (30 μM) treated paper strip after exposure to various vapors (DI H_2_O, N_2_H_4_, HN(CH)_2_, H_2_CO, HCl, and the mixture of H_2_CO and N_2_H_4_). The photos were taken after the exposure for 30 s.

The aforementioned in vitro assay results represent that **HyP-2** has the capability of hydrazine detection with high sensitivity and selectivity, and a fast-response time. As a first practical application, we applied **HyP-2** to the paper strip spray assay for the detection of hydrazine (Figure 7a). **HyP-2** grafted cellulose paper strip showed negligible fluorescence under UV light (commercial hand light, 365 nm) (Figure 7b). When the paper strip was soaked in hydrazine solution, a bright blue fluorescence became visible on the paper within 30 s, and it was distinguishable under UV light. In the sensing ability towards vaporized hydrazine test, the **HyP-2** grafted paper strip showed a fluorescence response only in hydrazine containing sets, while the other vaporized organic compounds did not respond; dimethylamine (HN(CH)_2_), formaldehyde (H_2_CO), hydrogen chloride (HCl), and mixture of hydrazine/formaldehyde (Figure 7c). In the case of dimethylamine, green fluorescence was observed, which is correlated with naphthaldehyde intermediate generation (compound **3**, **HyP-1**). We have an ongoing project that relates to this secondary amine sensing result.

### 3.5. Hydrazine Sensing Application in Soils

**Figure 8:**
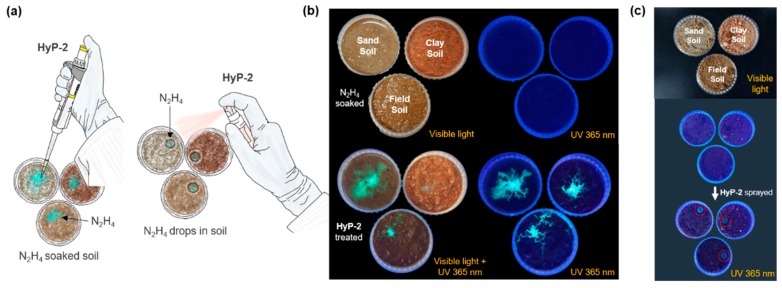
Soil application of **HyP-2**. (**a**) A schematic illustration for the hydrazine sensing test in various soil samples. (**b**,**c**) Photos of N_2_H_4_ moistened soils before and after treatment of **HyP-2,** under natural and UV light (365 nm, 3W and 6W). **HyP-2** (30 μM) was sprayed 10 times. (**b**) Hydrazine solution (3 mL) was treated to the entire area of the soils (sand, clay, and field soil). (**c**) 4 μL of **HyP-2** (30 μM) was dropped at specific points (red circle).

To explore the applicability of **HyP-2** in environmental analysis, we performed a real time detection of hydrazine in various soils (Figure 8). The hydrazine sensing test result within different soils (sand, clay, and field soil) suggested that **HyP-2** sense hydrazine (N_2_H_4_) specifically and selectively within any of the soils. In our previous system, **HyP-1**, it was difficult to distinguish the hydrazine exposure within soils under UV light because of the ratiometric responses. However, with **HyP-2,** it was possible to distinguish the presence or absence of hydrazine by simply monitoring the fluorescence under UV irradiation with its turn-on fluorescence response. We adopted two different types of screening methods; [Type 1] The solution of **HyP-2** (4 μL, 30 μM in DI H_2_O) was dropped on the pretreated-hydrazine (100 mM in DI H_2_O, 3 mL) soils (~ 1g) (Figure 8a, left). [Type 2] The **HyP-2** solution (30 μM in DI H_2_O) was sprayed (10 times) on the dry soils, which had pretreated-hydrazine soils (100 mM in DI H_2_O, 100 μL) in specific areas (Figure 8a, right). The fluorescence changes were immediately monitored at room temperature (25 °C) using UV hand light (365 nm). We observed a bright blue fluorescence from all soil samples within 1 min, and we also monitored the hydrazine distribution (Figure 8b). In the spray-based hydrazine sensing application, the fluorescence response was only observed in the spot of pretreated-hydrazine soils (Figure 8c, red circle). These results prove that the superior sensing ability of **HyP-2** in raw environmental conditions has great potential for further practical applications across various fields.

### 3.6. Hydrazine Sensing Application in Real Water Samples

**Figure 9:**
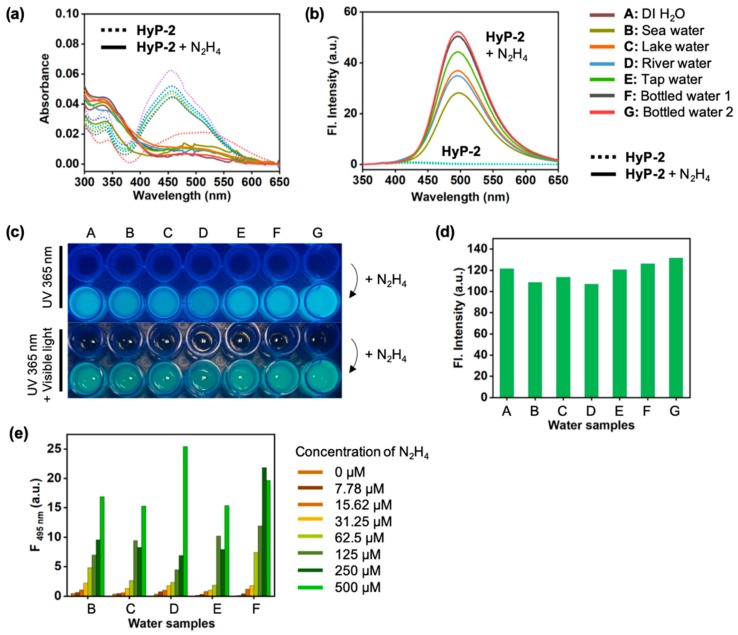
Detection of N_2_H_4_ in real water samples. (**a**) Absorption and (**b**) emission spectra changes of **HyP-2** (10 μM) after adding N_2_H_4_ (1 mM) in various real water samples, analyzed after incubating for 60 min at 25 °C. The emission spectra were obtained under excitation at the maximum wavelength of absorption. (**c**) A photo of **HyP-2** (upper, 10 μM) and **HyP-2** with N_2_H_4_ (bottom, 1 mM) in various water samples under UV light (365 nm) and visible light. The photo was taken after 60 min incubation at 25 °C. (**d**) Fluorescent intensity plot from solutions as shown in panel (c). The relative intensity was calculated using ImageJ software (NIH, Bethesda, USA). (**e**) Fluorescent intensity plot (at 495 nm) of **HyP-2** (10 μM) with various concentration of N_2_H_4_ (0–500 μM) within water samples. The intensity was recorded after 60 min incubation at 25 °C.

To evaluate whether the **HyP-2** could detect hydrazine in real environmental samples, the **HyP-2** was tested in experiments within the various samples; lake water, river water, sea water, tap water, and commercial bottled drinking water (Figure 9, Table S3). The hydrazine was added into the water samples and then incubated with the **HyP-2** (10 μM) for 60 min at 25 °C. As shown in Figure 9, a significant fluorescence enhancement was observed in the all water samples and the turn-on factor was between 87 times (river water, pH 7) and 363 times (DI H_2_O, pH 7) (Table S3). Under UV light (365 nm), the fluorescence signal was clearly monitored (Figure 9c,d), and the concentration dependent assay results provided superior sensing ability of **HyP-2** towards hydrazine within the real water samples at a low concentration range (0–500 μM) (Figure 9e).

## 4. Conclusions

In conclusion, we disclosed a turn-on type of hydrazine-selective fluorescent probe, **HyP-2**, which has a working mechanism based on *o*-MOM, an assisted retro-aza-Henry type reaction with hydrazine. **HyP-2** shows a significant fluorescence enhancement at 495 nm, after reacting with hydrazine within multifarious environments, including vapors, soils samples, and various water samples. In comparison with a control compound, **HyP-2C** which has no *o*-MOM, **HyP-2** showed a stronger sensing ability towards hydrazine with a fast-response time and dramatic increment of fluorescence. **HyP-2** shows high selectivity and sensitivity (0.05 ppb), and a fast response (< 5 min) towards the hydrazine. We used the **HyP-2** as a real-time sensing kit in order to study the hydrazine sensing in environments samples, and it provided superiority within these practical applications. This is a new hydrazine sensing approach, and we believe it has the capability to serve as a useful sensor for hydrazine detection in various fields.

## Supplementary Materials

The following are available online at https://www.mdpi.com/1424-8220/19/20/4525/s1, Figure S1: Solvent-dependent absorption and emission changes of **HyP-2**. Figure S2: Concentration dependent absorption spectra of **HyP-2** (0–40 μM) in DI H_2_O. Figure S3: Determination of the fluorescence quantum yield (Q.Y.). Figure S4: ^1^H NMR peak analysis of **HyP-2** and **HyP-2**+N_2_H_4_. Figure S5: Sensing properties of **HyP-2**. Figure S6: pH-dependent absorption (top) and emission (bottom) spectra changes of **HyP-2** (10 μM) and **HyP-2C** (control compound, 10 μM) after adding hydrazine (1 mM). Figure S7: Photostability of **HyP-2**. Table S1: Summary of hydrazine probes based on dicyanovinyl molecular rotor moiety. Table S2: Photophysical properties of **HyP-2**. Table S3: Emission intensity values (at peak) of **HyP-2** (10 μM) and **HyP-2** with hydrazine (1 mM) in various real water samples.
