# Supplementary material for: Hydrazine-Selective Fluorescent Turn-On Probe Based on Ortho-Methoxy-Methyl-Ether (o-MOM) Assisted Retro-aza-Henry Type Reaction
Source: Sensors (Basel). 2019 Oct 17;19(20):4525. doi: 10.3390/s19204525 (PMC6832147; doi:10.3390/s19204525)
Supplement: Supplementary file 1 [file sensors-19-04525-s001.pdf]

SUPPLEMENTARY INFORMATION

Hydrazine-selective Fluorescent Turn-on Probe based on Ortho-methoxy-methyl-ether (o-MOM) Assisted Retro-aza-Henry Type Reaction

This includes:

Supporting Figures: Fig. S1 to S7

Supporting Tables: Table S1 to S3

^{1}H and ^{13}C NMR for **HyP-2**

High resolution mass spectra of **HyP-2**

References and notes

Supporting Figures

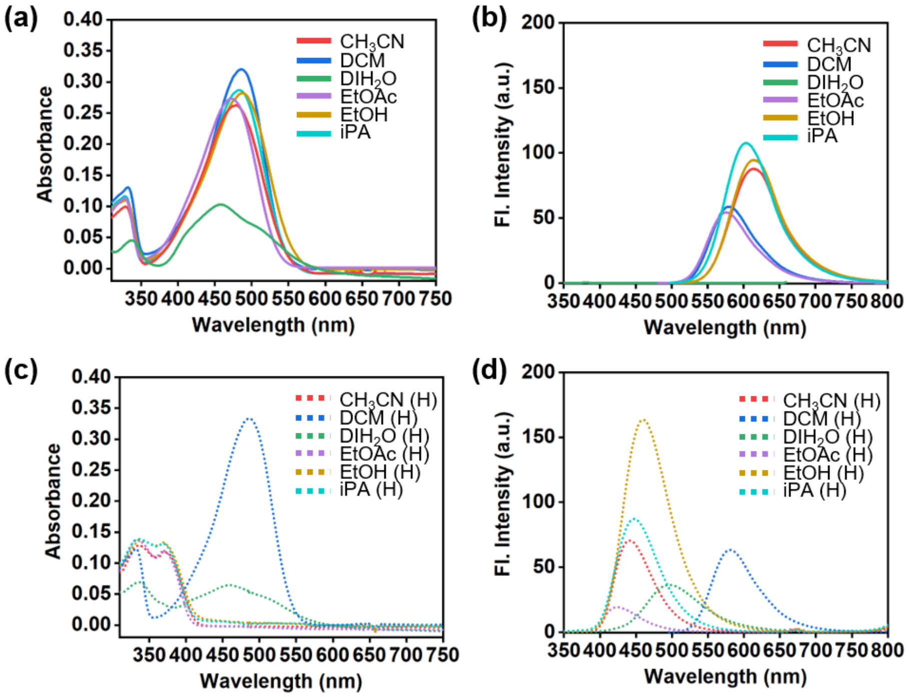

Figure S1. Solvent-dependent absorption and emission changes of **HyP-2**. (a, c) Absorption and (b, d) emission spectra of **HyP-2** (10 μM, top) and after adding hydrazine (1 mM, bottom) in various organic solvents, analyzed after 60 min incubation at 25 °C. The emission spectra were obtained under excitation at the maximum absorption wavelength within each solvent.

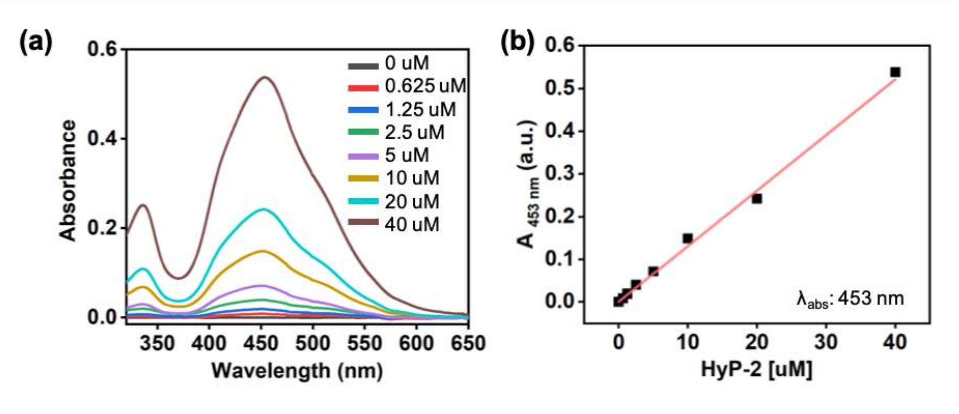

Figure S2. (a) Concentration dependent absorption spectra of **HyP-2** (0–40 μM) in DI H_2O. Absorption spectra was collected at 25 °C with no incubation. (b) absorbance intensity plot (peak height at 453 nm) of **HyP-2**, which is derived from the panel (a).

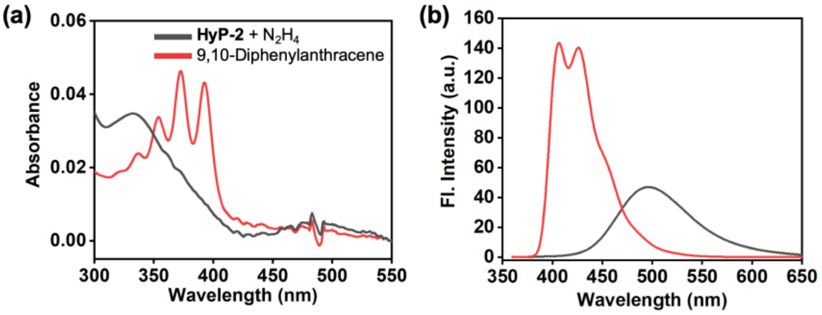

(c)

	9,10-Diphenylanthracene (ref)	HyP-2 + N_2H_4
Excitation (nm)	350	350
η	1.356 (EtOH)	1.333 (DIH_2O)
I	8,409,410	4,475,830
A	0.03002	0.02886
QY	0.88	0.47

Figure S3. Determination of the fluorescence quantum yield (Q.Y.). (a, b) Absorption and emission spectra of **HyP-2** (10 μM) + N_2H_4 (1 mM) in DI H_2O, and 9,10-diphenylanthracene (DPA) in ethanol. (c) Experimental parameters for determining the Q.Y. measurements of reaction product. The reaction product was prepared by incubating the mixture of **HyP-2** and N_2H_4 for 60 min at 25 °C.

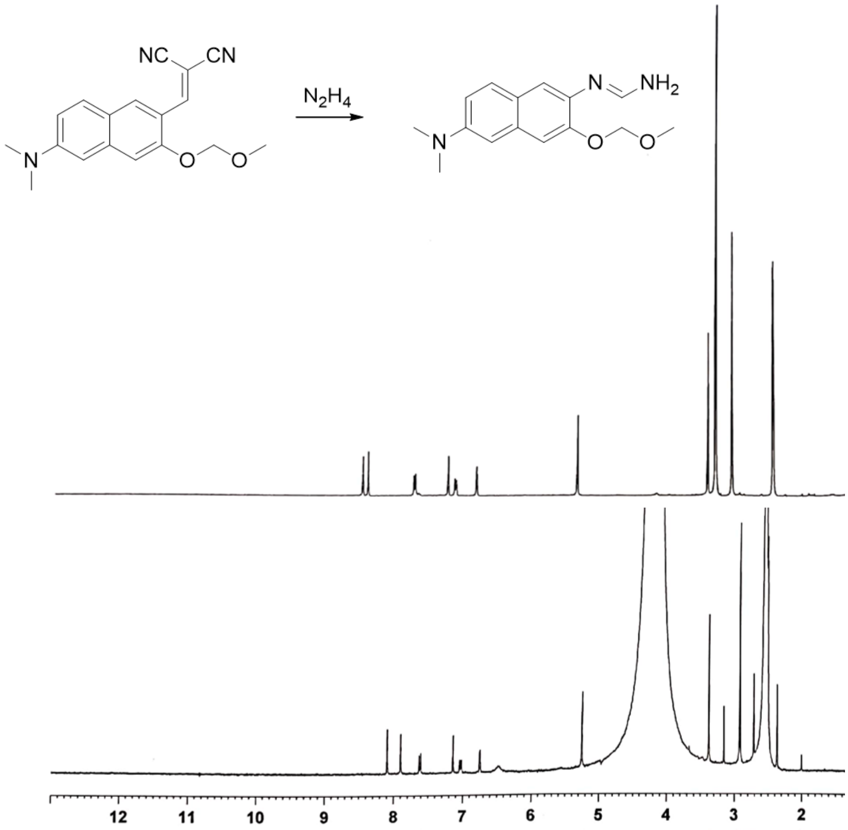

Figure S4. ^{1}H NMR peak analysis of **HyP-2** and **HyP-2**+N_2H_4. (a) ^{1}H NMR spectra of **HyP-2** (top, 3 mg/mL) and its reaction product (bottom) with N_2H_4 (crude solution in DI H_2O) in DMSO-d_6 NMR solvent. NMR tube with the mixture of **HyP-2** and N_2H_4 was incubated for 60 min at 25 °C.

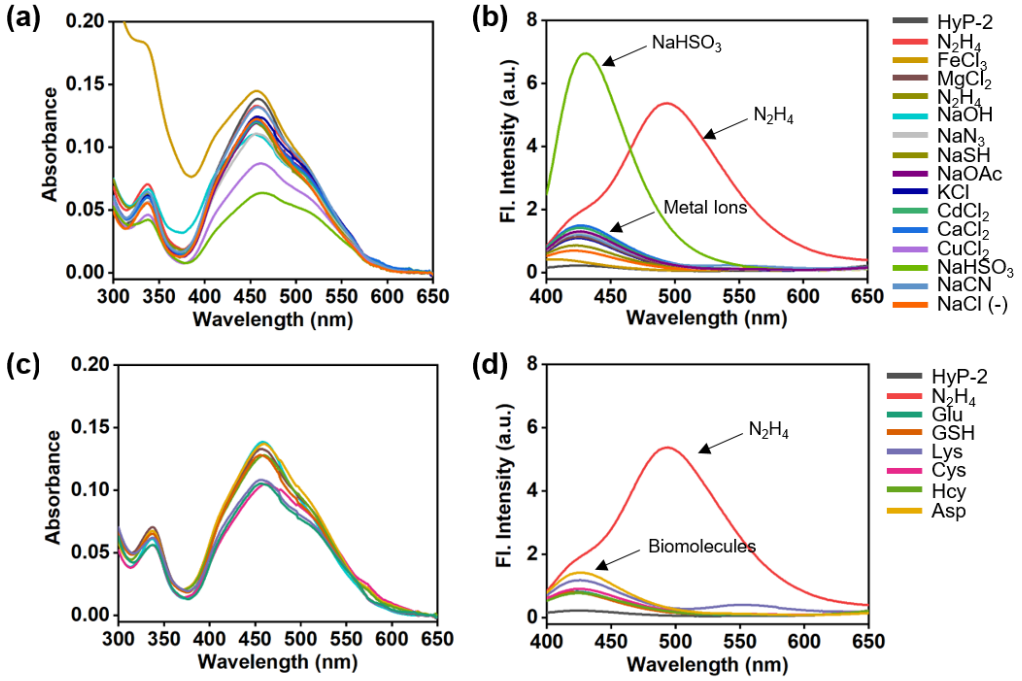

Figure S5. Sensing properties of **HyP-2**. (a, c) Absorption and (b, d) emission spectra of **HyP-2** (10 μM) after adding each metal ions (30 eq) and biomolecules (30 eq) including hydrazine solution (1 mM) in DI H_2O, measured after incubating for 60 min at 25 °C. [*Metal ions*] $CaCl_2$, $CdCl_2$, $CuCl_2$, $FeCl_3$, KCl, $MgCl_2$, NaCl, NaCl (anion), NaCN, $NaHSO_3$, NaN_3, NaOAc, NaOH, NaSH, $NiCl_2$, and $ZnCl_2$. [*Biomolecules*] Glu (glutamine), GSH (glutathione), Lys (lysine), Cys (cysteine), Hcy (homocysteine), and Asp (aspartic acid). The emission spectra were obtained under excitation at 338 nm.

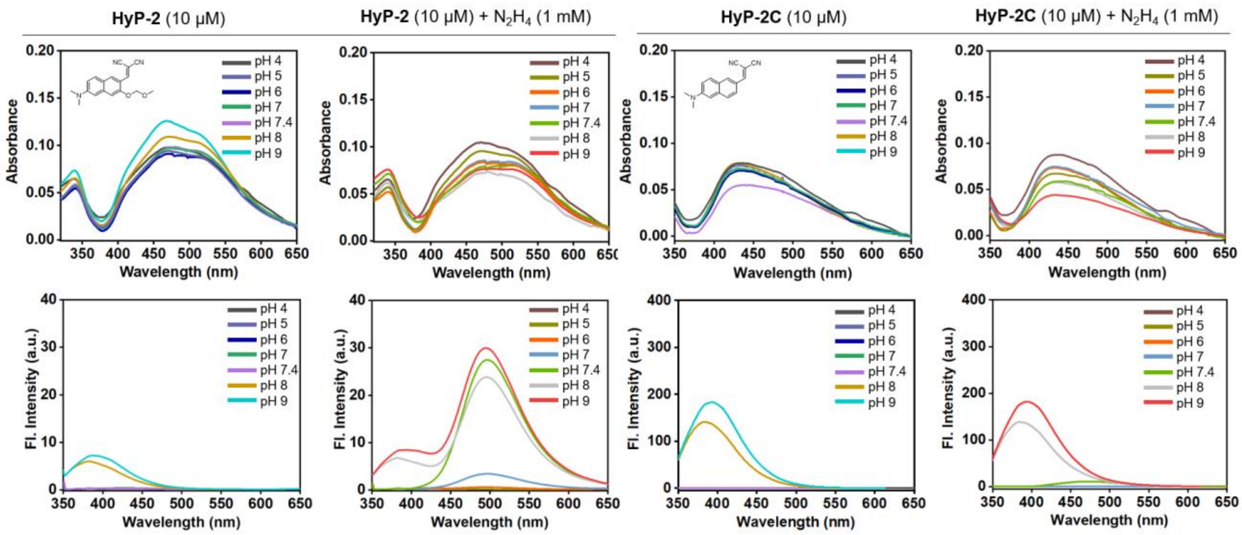

Figure S6. pH-dependent absorption (top) and emission (bottom) spectra changes of **HyP-2** (10 μM) and **HyP-2C** (control compound, 10 μM) after adding hydrazine (1 mM). Spectra changes were monitored in various pH buffers (pH 4, 5, 6, 7, 7.4, 8, 9), measured after incubating for 60 min at 25 °C. The fluorescence emission spectra were obtained under excitation at the maximum absorption wavelength.

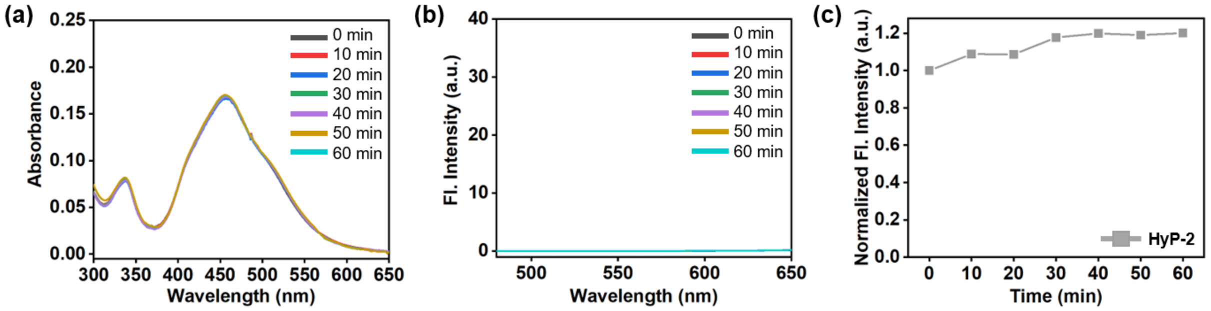

Figure S7. Photostability of **HyP-2**. (a) Absorption and (b) emission change of **HyP-2** (10 μM) under continuous UV light exposure (365 nm, 3 W) in DI H_2O. Time indicates the UV light exposure time. (c) A fluorescence intensity change plot, which is derived from the maximum wavelength of emission spectra in panel (b).

^{1}H NMR spectra for **HyP-2C**

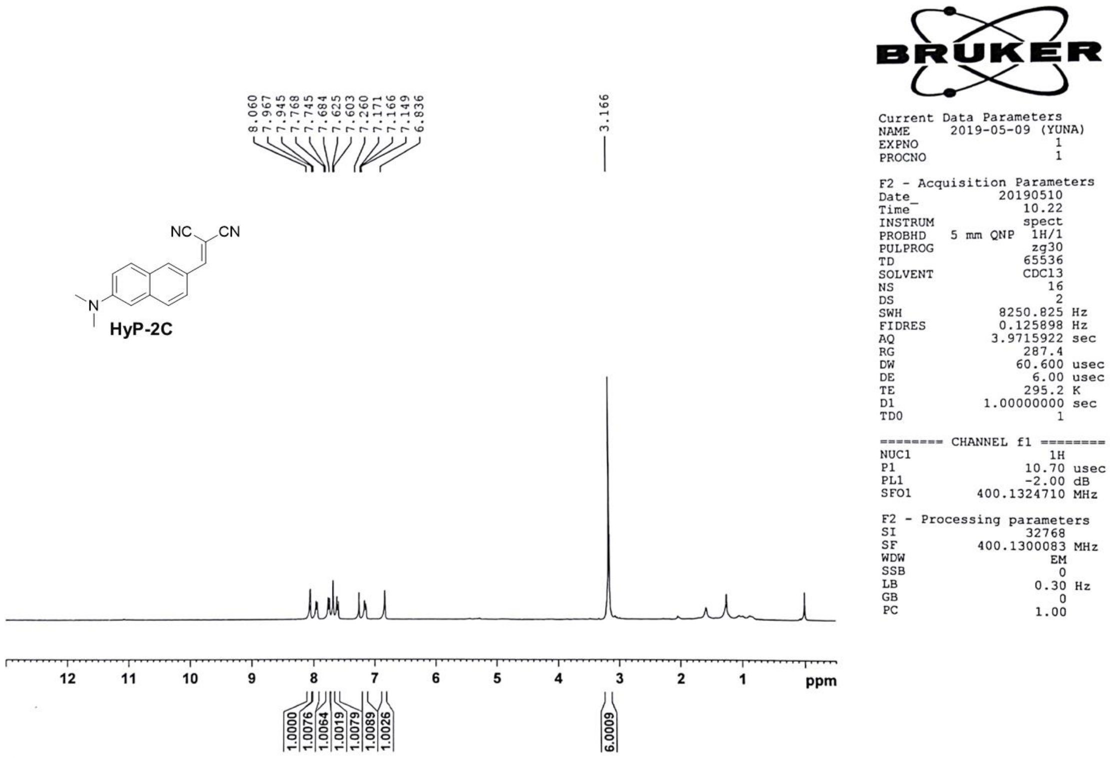

^{13}C NMR spectra for **HyP-2**

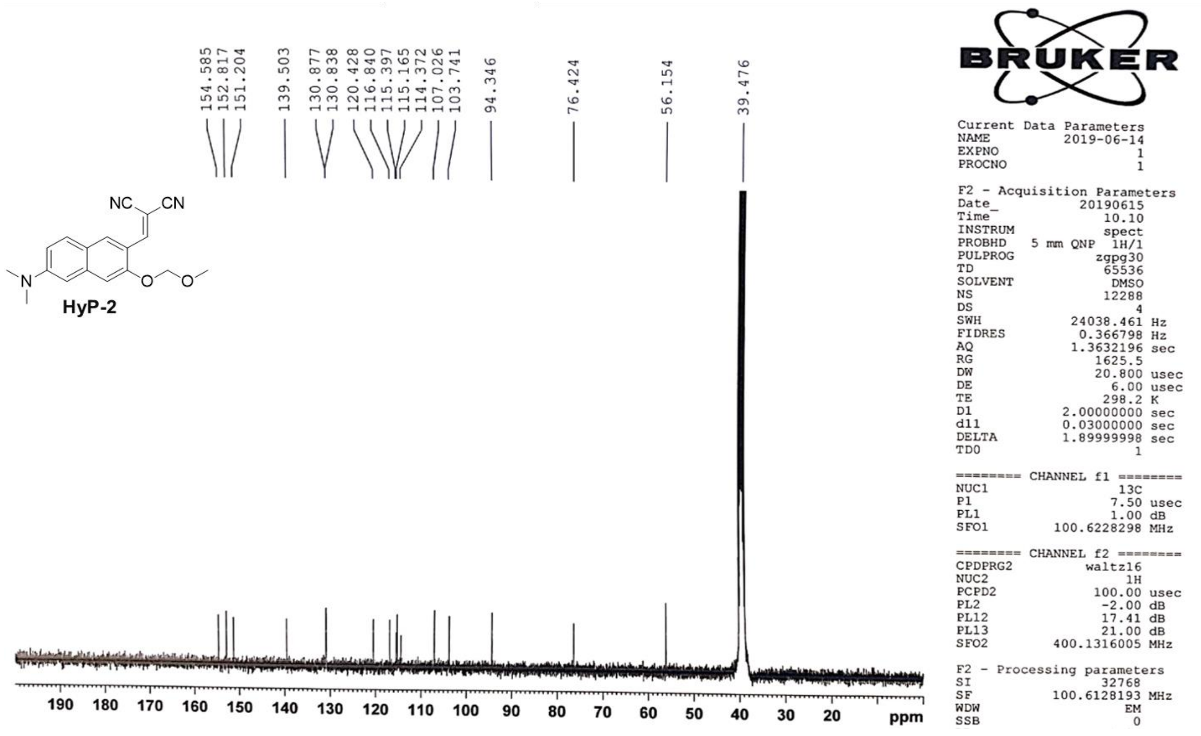

^{13}C NMR spectra for **HyP-2**+N_2H_4

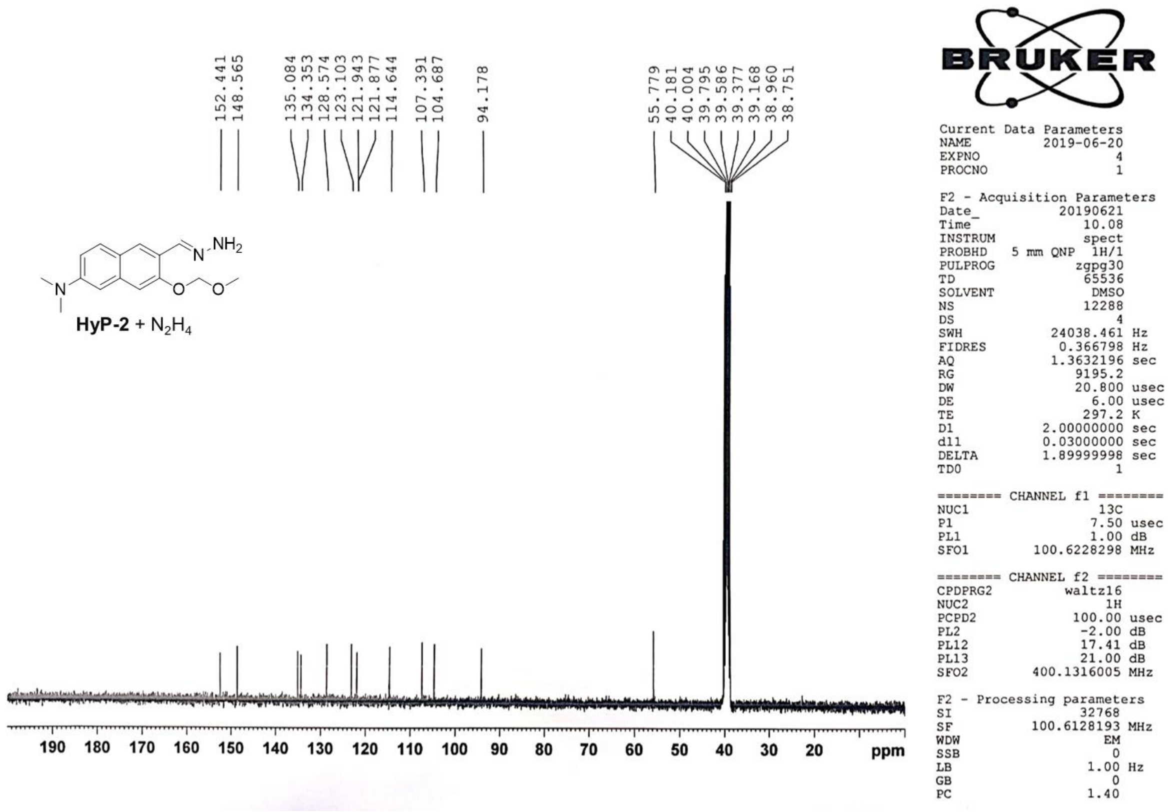

HR-mass spectra for **HyP-2**

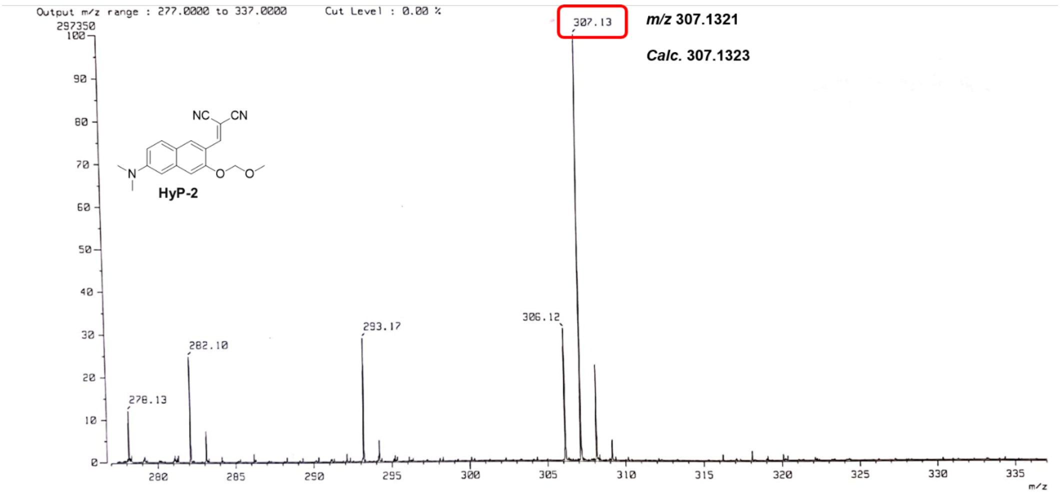

HR-mass spectra for **HyP-2**+N_2H_4

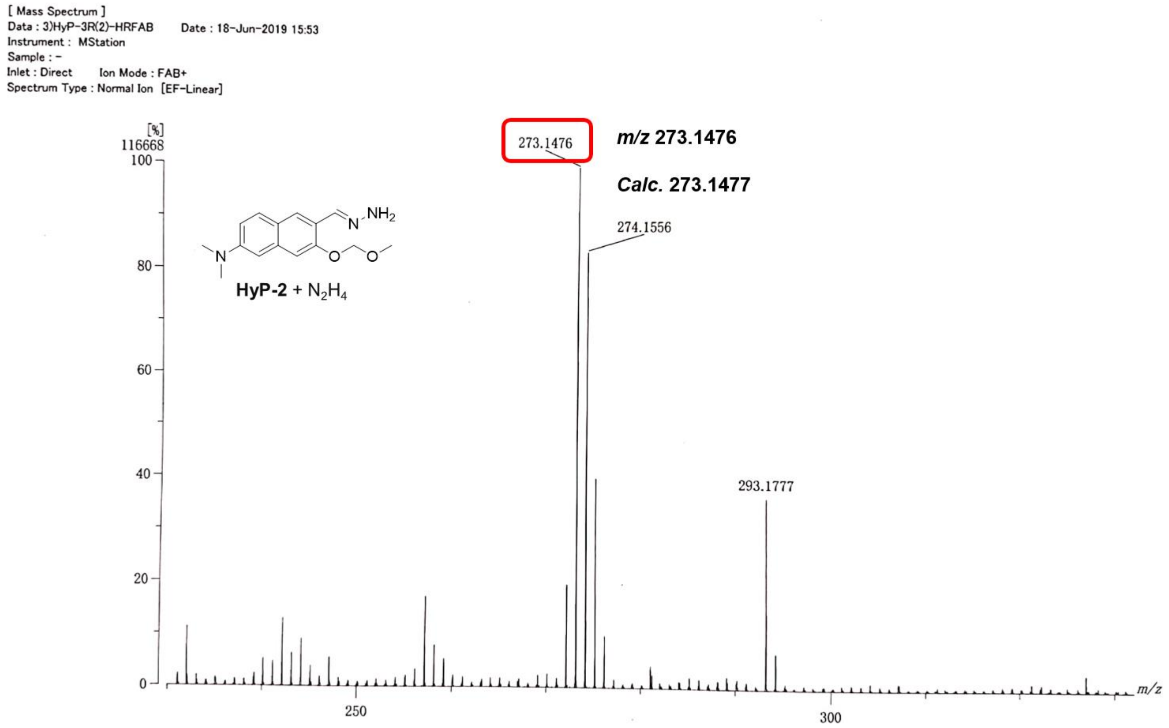

Supporting Tables

Table S1. Summary of hydrazine probes based on dicyanovinyl molecular rotor moiety. DMSO: dimethyl sulfoxide, CH_3CN: acetonitrile, THF: tetrahydrofuran, EtOH: ethanol.

Structure	Type	Sensitivity	Selectivity	Response time	Media	Application
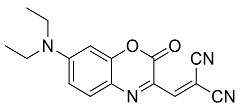<br>(Ref. 1)	Ratiometric<br>(λ_{exc} = 510 nm,<br>λ_{emi} = 639nm)	0.43μM	○	20 min	pH 3.7 buffer–DMSO<br>(1:9, *v/v*)	Cell imaging
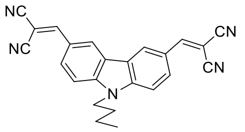<br>(Ref. 2)	Ratiometric<br>(λ_{exc} = 405 nm,<br>λ_{emi} = 458nm)	1.02 μM	○	< 1 min	pH 7.4 buffer-CH_3CN<br>(8:2, *v/v*)	Not Reported
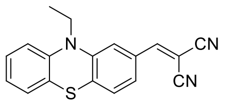<br>(Ref. 3)	Ratiometric<br>(λ_{exc} = 470 nm,<br>λ_{emi} = 495 nm)	121.91 μM.	○	Not Reported	pH 7.5 buffer-DMF<br>(7:3, *v/v*)	Cell imaging, zebra fish imaging, Paper strip
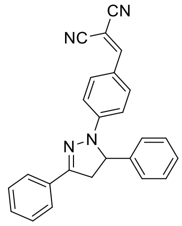<br>(Ref. 4)	Off-on<br>(λ_{exc} = 460 nm,<br>λ_{emi} = 520 nm)	6.16 μM	○	30 min	pH 5 buffer-CH_3CN<br>(9:1, *v/v*)	Not Reported
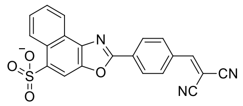<br>(Ref. 5)	Ratiometric<br>(λ_{exc} = 350 nm,<br>λ_{emi} = 400 nm)	1.79 nM	○	40 sec	pH 7.4 buffer	Cell imaging, Vapor test
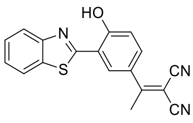(Ref. 6)	Off-on<br>(λ_{exc} = 440 nm,<br>λ_{emi} = 510 nm)	29 μM	○	55 min	DMSO-H_2O<br>(8:2, *v/v*)	Silica gel plate test, Cell imaging
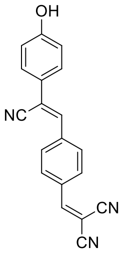<br>(Ref. 7)	On-off<br>(λ_{exc} = 370 nm,<br>λ_{emi} = 635 nm)	3.67 μM	○	Not Reported	THF-H_2O<br>(2:8, *v/v*)	Cell imaging, Paper strip

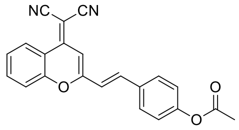 (Ref. 8)	Off-on (λ_{exc} = 551 nm, λ_{emi} = 680 nm)	570 μM	○	1 min	pH 7.4 buffer-EtOH (7:3, *v/v*)	Cell imaging, Paper strip
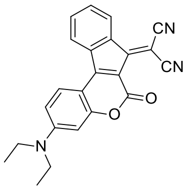 (Ref. 9)	Off-on (λ_{exc} = 638 nm, λ_{emi} = 692 nm)	8.6 nM	○	30 min	pH 7.4 buffer-CH_3CN (5:5, *v/v*)	Cell imaging, Paper strip
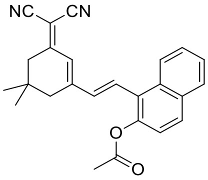 (Ref. 10)	Off-on (λ_{exc} = 691 nm, λ_{emi} = 725 nm)	0.0823 μM	○	Not Reported	DMSO	Paper strip

Table S2. Photophysical properties of **HyP-2**. ACN; acetonitrile, DCM; dichloromethane, DI H_2O; deionized water, EtOAc; ethyl acetate, EtOH; ethanol, iPrOH; isopropyl alcohol.

Compound	Solvent	λ_{abs} (nm)	λ_{fl} (nm)	Stoke's shift
**HyP-2**	ACN	486	614	128
	DCM	487	579	92
	DI H_2O	459	711	42
	EtOAc	471	576	105
	EtOH	488	613	125
	iPrOH	487	603	116

Table S3. Emission intensity values (at peak) of **HyP-2** (10 μM) and **HyP-2** with hydrazine (1 mM) in various real water samples. The values were recorded after 60 min incubation at 25 °C. The emission intensity was obtained under excitation at the maximum wavelength of absorption.

Water Samples	HyP-2	HyP-2 + N_2H_4	Turn-on factor
DI H_2O (pH 7)	138.88	50506.84	363 times
Sea water (pH 8)	281.01	28105.58	100 times
Lake water (pH 6)	347.46	36875.32	106 times
River water (pH 7)	397.82	34921.48	87 times
Tap water (pH 7)	177.79	44282.26	249 times
Bottled water 1 (pH 8)	164.99	50319.63	304 times
Bottled water 2 (pH 6.5)	178.99	52194.9	291 times

References and Note

References for Table S1

1. [1] J.L. Fan, W. Sun, M.M. Hu, J.F. Cao, G, H. Cheng, H.J. Dong, K.D. Song, Y.C. Liu, S.G.
2. Sun and X. J. Peng, *Chem. Commun.*, 2012, **48**, 8117–8119.
3. [2] S. Goswami, S. Paul, A. Manna, *RSC Advances*, 2013, **3**,18872-18877.
4. [3] M. Sun, J. Guo, Qingbiao Yang, N. Xiao, Y. Li, *J. Mater. Chem. B*, 2014, **2**,1846-1851.

5. [4] X. Zheng, S. Wang, H. Wang, R. Zhang, J. Liu, B. Zhao, *Spectrochim. Acta A*, 2015, **138**, 247–251.
6. [5] Shweta, A. Kumar, Neeraj, S.K. Asthana, A. Prakash,J. K. Roy, I. Tiwaria, K.K. Upadhyay, *RSC Adv.*, 2016, **6**, 94959–94966.
7. [6] Z. Chen, X. Zhong, W. Qu, T. Shi, H. Liu, H. He, X. Zhang, S. Wang, *Tetrahedron Letters*, 2017, **58**, 2596–2601.
8. [7] J. Qiu, Y. Chen, S. Jiang, H. Guo, F. Yang, *Analyst*, 2018, **143**, 4298–4305.
9. [8] J. Ma, J. Fan, H. Li, Q. Yao, J. Xia, J. Wang, X. Peng, *Dyes Pigm.*, 2017, **138**, 39–46.
10. [9] Y. Liua, D. Rena, J. Zhanga, H. Lib, XF. Yanga, *Dyes Pigm.*, 2019, **162**, 112–119.
11. [10] X. Shi, F. Huo, J. Chao, Y. Zhanga, C. Yin, *New J. Chem.*,2019,**43**, 10025-10029.
